# Translational genomics for achieving higher genetic gains in groundnut

**DOI:** 10.1007/s00122-020-03592-2

**Published:** 2020-04-23

**Authors:** Manish K. Pandey, Arun K. Pandey, Rakesh Kumar, Chogozie Victor Nwosu, Baozhu Guo, Graeme C. Wright, Ramesh S. Bhat, Xiaoping Chen, Sandip K. Bera, Mei Yuan, Huifang Jiang, Issa Faye, Thankappan Radhakrishnan, Xingjun Wang, Xuanquiang Liang, Boshou Liao, Xinyou Zhang, Rajeev K. Varshney, Weijian Zhuang

**Affiliations:** 1 International Crops Research Institute for the Semi-Arid Tropics (ICRISAT), Hyderabad, India; 2 University of Southern Queensland (USQ), Toowoomba, Australia; 3 Central University of Karnataka, Gulbarga, India; 4 Mars-Wrigley, Chicago, USA; 5 Crop Protection and Management Research Unit, United State Department of Agriculture – Agricultural Research Service (USDA-ARS), Tifton, USA; 6 Peanut Company of Australia (PCA), Kingaroy, Australia; 7 University of Agricultural Sciences (UAS), Dharwad, India; 8 Crops Research Institute (CRI), Guangdong Academy of Agricultural Sciences (GAAS), Guangzhou, China; 9 ICAR-Directorate of Groundnut Research (DGR), Junagadh, India; 10 Shandong Peanut Research Institute (SPRI), Qingdao, China; 11 Oil Crops Research Institute (OCRI), Chinese Academy of Agricultural Sciences (CAAS), Wuhan, China; 12 Institut Sénégalais de Recherches Agricoles (ISRA)-Centre National de Recherches Agronomiques (CNRA), Bambey, Senegal; 13 Biotechnology Research Center, Shandong Academy of Agricultural Sciences (SAAS), Jinan, China; 14 Henan Academy of Agricultural Sciences (HAAS), Zhenzhou, China; 15 Institute of Oil Crops, Fujian Agriculture and Forestry University, Fuzhou, 350002 China

## Abstract

**Key message:**

Groundnut has entered now in post-genome era enriched with optimum genomic and genetic resources to facilitate faster trait dissection, gene discovery and accelerated genetic improvement for developing climate-smart varieties.

**Abstract:**

Cultivated groundnut or peanut (*Arachis hypogaea*), an allopolyploid oilseed crop with a large and complex genome, is one of the most nutritious food. This crop is grown in more than 100 countries, and the low productivity has remained the biggest challenge in the semiarid tropics. Recently, the groundnut research community has witnessed fast progress and achieved several key milestones in genomics research including genome sequence assemblies of wild diploid progenitors, wild tetraploid and both the subspecies of cultivated tetraploids, resequencing of diverse germplasm lines, genome-wide transcriptome atlas and cost-effective high and low-density genotyping assays. These genomic resources have enabled high-resolution trait mapping by using germplasm diversity panels and multi-parent genetic populations leading to precise gene discovery and diagnostic marker development. Furthermore, development and deployment of diagnostic markers have facilitated screening early generation populations as well as marker-assisted backcrossing breeding leading to development and commercialization of some molecular breeding products in groundnut. Several new genomics applications/technologies such as genomic selection, speed breeding, mid-density genotyping assay and genome editing are in pipeline. The integration of these new technologies hold great promise for developing climate-smart, high yielding and more nutritious groundnut varieties in the post-genome era.

## Introduction

Groundnut or peanut (*Arachis hypogaea*) is one of the most nutritious oilseed and legume crop, grown in > 100 countries of tropics and subtropics regions of the world. Currently, this crop is cultivated globally in over 28.5 million hectares which yielded 45.95 million tons of pods during 2018 (FAOSTAT 2018). The Asia (40.2%) and Africa (54.9%) regions hold together 95% of global groundnut cultivated area with the annual production contribution of 59.3% and 31.1%, respectively. All plant parts of groundnut are useful and are major sources of nutrition for both humans and livestock. Groundnut is popularly known as poor man’s almonds for its high nutritional content with fat and protein making up 80% of seeds contents and is therefore a key contributor in the fight against malnutrition. More importantly, groundnut is an important ingredient in hundreds of delicious preparations and commercial products (see Pandey et al. 2012; Pandey and Varshney 2018). Groundnut is also a rotation crop which enriches soil fertility through nitrogen fixation and breaking disease and pest cycles.

Diversification of food habits and frequent migration of population across the globe have greatly helped in bringing awareness about the varied modes of groundnut consumption. For example, this crop is consumed as an edible food crop in western countries, while the major portion of the groundnut production is used for oil extraction in most of the Asian countries. Also, the industry players have introduced several groundnut-based food products creating bigger consumer markets on a global basis. Several groundnut-based nutritional supplements are being produced and distributed to malnutrition-affected regions in Africa and Asia. Currently, groundnut-based food consumption has drastically increased across the globe, more in Asia and Africa, suggesting groundnut may become a primary food crop rather than an oilseed crop in the coming decades, especially with development of high oleic groundnut lines. The responsibility is, therefore, now on the groundnut researchers to keep groundnut improving using different modern genomic tools to strengthen its position in both oilseed and food applications.

Increasing the pod yield and oil content in addition to improving resistance/tolerance to various biotic and abiotic stresses has been the core objectives of all the groundnut breeding programs across the globe. The recent climate change conditions may further pose difficulties before these researchers in developing new varieties for wider adoptions. In addition, the breeders need to develop new varieties with preferred traits of farmers, industry and consumers such as high oleic acid, and kernel features to ensure wider adoption and acceptance of new varieties and financial benefits to the farmers/growers. Modern genomics hold great promise in accelerating the process of trait mapping, candidate gene discovery, functional gene identification, marker development and molecular breeding (see Varshney et al. 2013, 2019; Pandey et al. 2016). The groundnut research community has developed these genomic resources and high-resolution genetic populations in a very short time over recent years. This article reviews the current status and strategies for developing and deploying genomic resources and tools for groundnut improvement for developing climate-smart and consumer-preferred varieties.

## Genetic resources for high-resolution mapping

Genetic diversity in a given crop is the core asset for further crop improvement, and therefore, genetic resources hold the path to success in enhancing the genetic gain and quality of the production in farmers’ field. The groundnut crop is blessed with large germplasm collections maintained in India (15,445 accessions), USA (9310 accessions) and China (7837 accessions) (see Pandey et al. 2012). The genera *Arachis* can be divided into nine sections consisting of 81 species from two ploidy groups (diploid and tetraploid) with huge diversity of genomes (A, B, AB, D, F, K, EX, T, PR, H, C, T, E, R_1_ and R_2_) (see Stalker 2017). Of these 81 species, the *A. hypogaea* species has more relevance as it is the only cultivated species that is grown commercially today in the field. These germplasm collections have been characterized for important agronomic traits and have different diversity panels for use in breeding. For example, the ICRISAT, USA and China initially developed core collection (with 1704, 831 and 576 genotypes, respectively) which were further reduced in size for easy management leading to development of minicore collection (with 184, 112 and 298 genotypes, respectively). In addition, more panels such as composite collection and reference set were also developed by ICRISAT to bring in more diversity which are now a very good resource for trait discovery and breeding (see Pandey et al. 2012). These diverse panels should be evaluated in multiple hot spot locations for targeting current and future traits of importance for use in marker discovery and crop breeding. Resequencing entire germplasm collections together with precise phenotyping data for currently and future need-based features may further help in redefining different diversity sets for varied applications in groundnut research and genetic improvement.

The initial trait mapping efforts identified markers linked to nematode resistance which were successfully deployed for improving the nematode resistance (Church et al., 2000) followed by gene discovery for high oleic trait (Chu et al 2009). The efforts gained fast momentum from 2009 onward in cultivated groundnut after the development of the first SSR-based genetic map based on RIL population (Varshney et al. 2009). Despite the major limitation of biparental population in its ability to tackle one or two traits in one go, such populations have been used in multiple studies for mapping several traits including foliar disease resistance, oil content and quality, aflatoxin contamination, yield and seed features (see Vishwakarma et al. 2017a). To address the above issues, multi-parent genetic populations, namely NAM (nested-association mapping) and MAGIC (multi-parent advanced generation intercross) populations, are being developed in groundnut segregating for multiple traits. In addition to several agronomic traits, the focused multi-parent populations have also been developed for complex traits such as drought tolerance and aflatoxin contamination (see Pandey et al. 2019a). For performing high-resolution genetic mapping, the mating design, frequency of functional marker alleles and their extent of genetic effects together with disequilibrium between functional and non-functional markers are very important in addition to implementation of appropriate statistical and genetic analytical methods (Guo et al. 2016; Wang et al. 2017). The NAM and MAGIC populations provide opportunities to bring recombination in higher frequency which makes the genetic population more diverse, an ideal condition for high-resolution trait mapping.

Two NAM populations (one each for Spanish and Virginia types) and three MAGIC populations (for drought, aflatoxin and multiple agronomic, disease and nutrition traits) have been developed at ICRISAT keeping in mind the diversity for agronomically important traits among founder parents (see Pandey et al. 2016). For developing NAM-Spanish, the Spanish bunch genotype, ICGV 91114, was crossed with 22 diverse genotypes, while Virginia bunch genotype, ICGS 76, was crossed with 21 testers to develop NAM–Virginia population. The first MAGIC population ‘MAGIC-Multiple traits’ was developed by crossing eight parental genotypes (ICGV 88145, ICGV 00308, ICGV 91114, ICGV 06040, ICGV 00440, ICGV 05155, GPBD 4 and 55–437) targeting multiple traits including fresh seed dormancy, oil content, seed mass, kernel Fe and Zn content, stem rot tolerance, aflatoxin contamination and PBND tolerance. The second MAGIC population ‘MAGIC-Aflatoxin’ was developed to bring in diversity and recombination for three resistance mechanisms for A.* flavus* infection and aflatoxin contamination (55–437, ICG 51, ICGV 12014, U4-7-5, VRR245, ICGV 88145, ICGV 89104 and ICGV 91278). The third MAGIC population ‘MAGIC-Drought tolerance’ targeted different component traits of drought tolerance (ICGV 02022, ICG 7190, ICGV 97183, ICG 3053, ICG 14482, ICG 11515, TAG 24 and ICGV 02266). Similarly, the USDA-ARS and the University of Georgia (UGA) developed two NAM populations, namely ‘NAM_Florida’ and ‘NAM_Tifrunner,’ using Florida-07 and Tifrunner as common parents and crossing with four founder parental genotypes (N08082, SPT06-06, C76-16 and NC3033). These NAM populations segregate for several agronomic traits in addition to resistance to tomato spotted wilt virus (TSWV), aflatoxin resistance, drought tolerance, early and late leaf spot resistance (Holbrook et al. 2013; Chu et al. 2018, 2019). In USA, a MAGIC population was also developed from eight founders: SunOleic 97R, NC94022, Tifrunner, GT-C20, Florida 07, SPT06-06, Georgia 13 M and TifNV-High O/L. These parental selections were based on the availability of genetic and genomic information to maximize genetic diversity while meeting practical breeding objectives including high oleic content (Guo et al. 2018).

## Genomic resources and tools in *Arachis*: journey from a resource-poor to resource-rich crop

**Fig. 1:**
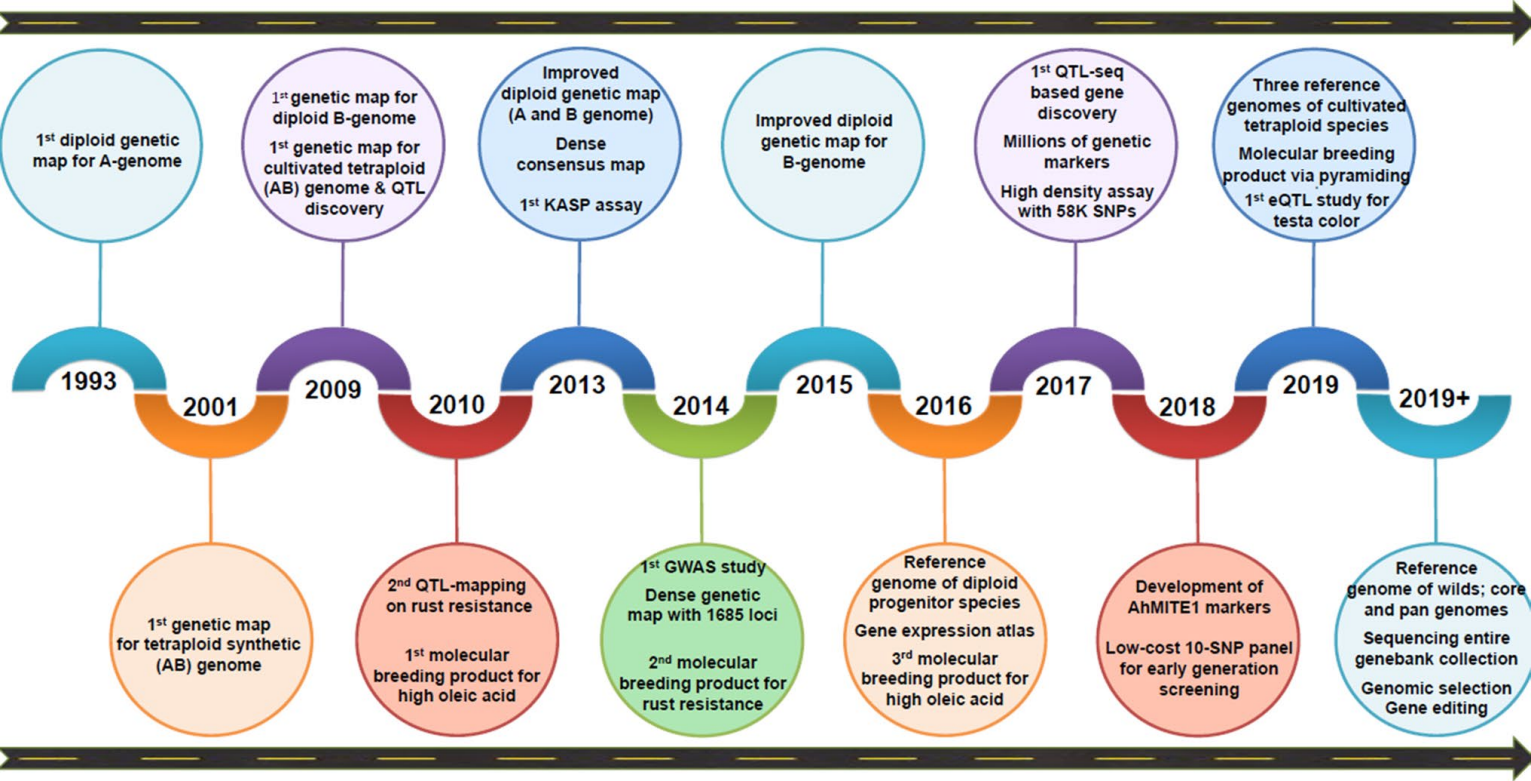
A milestone roadmap representing the important events in the area of groundnut genomics and molecular breeding

**Fig. 2:**
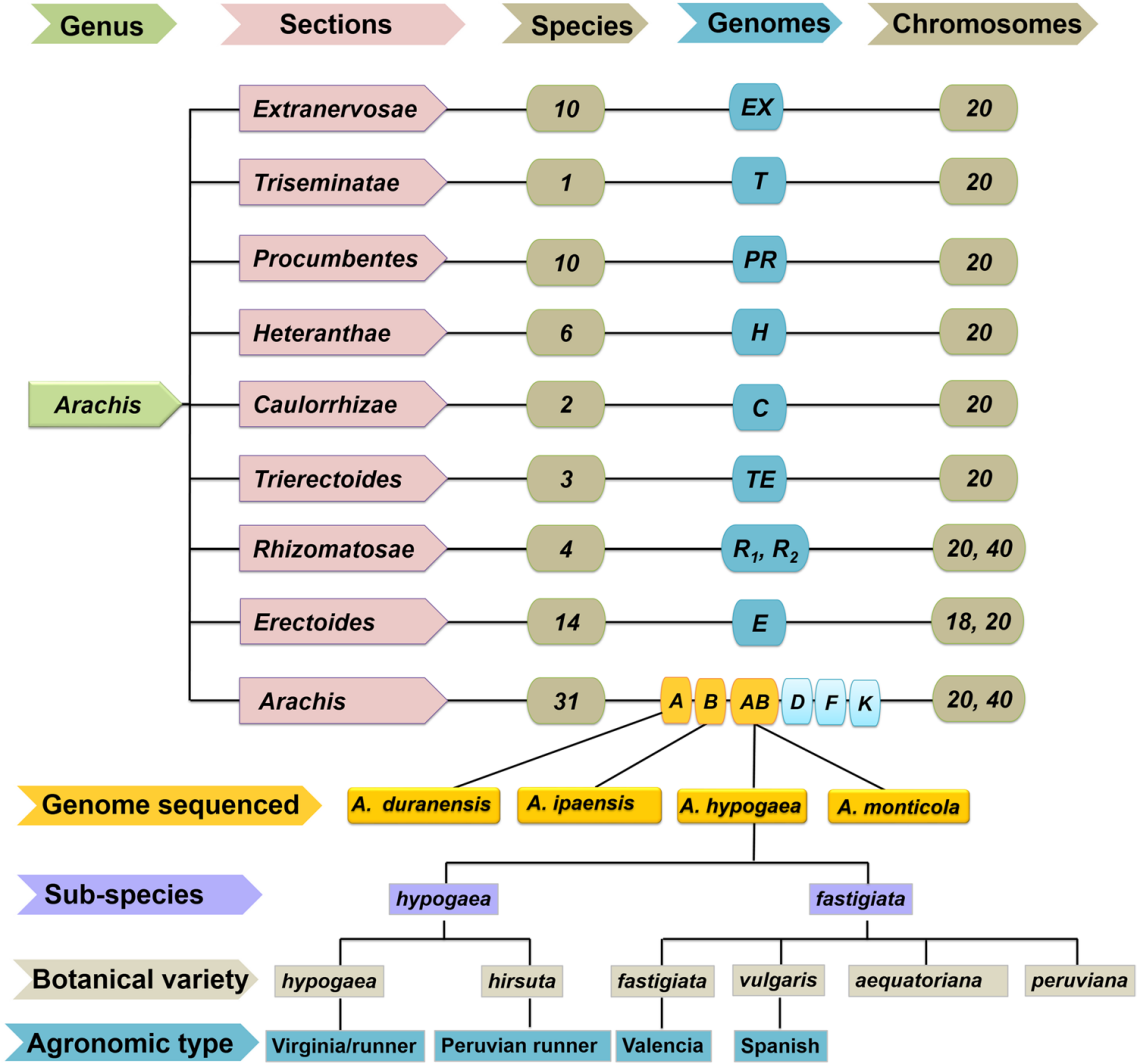
Species, chromosome (2n) and genome diversity in Arachis genus and genome sequencing

**Fig. 3:**
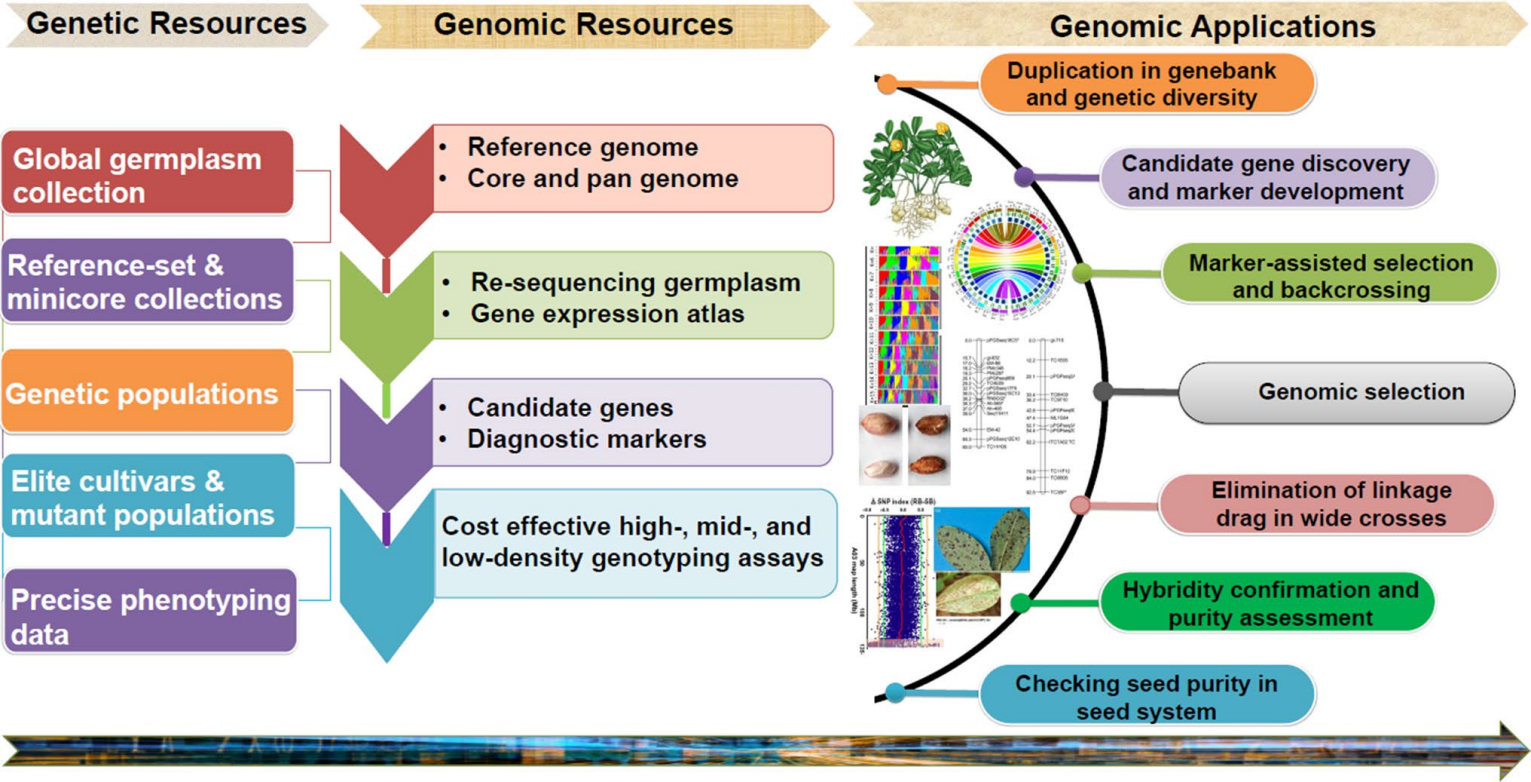
Application of marker technology from seed (germplasm) to seed (improved variety) in groundnut. The germplasm resource and the genetic variation are key for the development of the genetic resources and genetic/molecular markers. The development of molecular markers require huge efforts including development and assessment of biparental or complex mapping population along with high-throughput genotyping of diverse germplasm collections. The developed genetic markers can be used in various purposes for enhancing breeding efficiency such as molecular breeding product development as well as in ensuring genetic purity in seed chain

Groundnut research community has come a long way in achieving optimum genomic resources, more in last one decade (Fig. 1). Of the 15 different genomes (A, B, AB, D, F, K, EX, T, PR, H, C, T, E, R_1_ and R_2_) in *Arachis* genus, reference genomes have become available only for three (A, B, AB) genomes (Fig. 2). Genomic resources such as reference genomes, genome-wide genetic markers, genome-wide gene expression atlas, genotyping assays (low, mid and high density), quality control (QC) panels and diagnostic markers are very important for implementing translational genomics activities and different applications in agriculture (Fig. 3). Due to large genome size and allopolyploidy, the first-generation sequencing approaches lacked generation of longer reads which further created complexity in assembling in addition to being too expensive to initiate sequencing and developing reference genomes. The scientific revolution in the last two decades provided an array of sequencing technologies for high-throughput short reads, making assembling little easier with greatly reduced cost. The last decade has been a golden period for groundnut as the majority of the genomic resources were developed and have become available for the international community. These resources included reference genomes (for progenitors, wild tetraploid and cultivated tetraploid), genome-wide genetic markers, genome-wide gene expression atlases, high-density genotyping assays and diagnostic markers. Also, there are still many resources that are needed, which will become the next priority for this community to develop in coming years. These resources include reference genomes for wild diploids, high-throughput genotyping assays (low and mid density), functional genomics, quality control (QC) panels and diagnostic markers.

### Reference genomes

High-quality genome assembly and well-annotated genome are very crucial for its use in developing and deploying genomics tools for accelerated crop improvement. The reference genome plays important role in precise understanding of genome architecture, faster gene discovery and development of diagnostic/functional genetic markers. Since 2016, the reference genomes have become available for both the wild progenitors (diploids and tetraploid) and cultivated tetraploid groundnut (Table 1, Fig. 1). The International Peanut Genome Initiative (IPGI) through Peanut Genome Consortium (PGC) completed the sequencing of both the diploid progenitor species, namely *A. duranensis* V14167 (A genome) and *A. ipaensis* K30076 (B genome) (Bertioli et al. 2016). In parallel effort, the Diploid Progenitor Peanut A-Genome Sequencing Consortium (DPPAGSC) completed the sequencing of both the diploid progenitor species, i.e., *A. duranensis* PI475845 (A genome; Chen et al. 2016) and *A. ipaensis* ICG 8206 (B genome; Lu et al. 2018). The IPGI-led assembled genome assemblies were preferred for performing the genomics studies related to comparative genomics, transcriptomes and sequence-based trait mapping and marker development. These genomes predicted 1.2 Gb genome size for *A. duranensis* (36,734 genes) and 1.5 Gb genome size for *A. ipaensis* (41,840 genes) making total to the estimated genome size for cultivated groundnut (~ 2.7 Gb). In 2018, the genome assembly has been made available for the allotetraploid wild groundnut (~ 2.62 Gb; 20 pseudomolecules), *A. monticola* PI 263,393, which is considered either the direct progenitor for the cultivated tetraploid groundnut or as an independent derivative between the cultivated groundnut and wild species (Yin et al. 2018). This study even deployed highly sophisticated and advanced technologies such as Hi-C technology to develop this high-quality genome assembly for the wild tetraploid.

**Table 1:** Summary of genome sequence information for diploid progenitors, wild tetraploid and cultivated groundnut

Species	*A. duranensis*	*A. duranensis*	*A. ipaensis*	*A. ipaensis*	*A. monticola*	*A. hypogaea* subsp. *hypogaea*	*A. hypogaea* subsp. *fastigiata*	*A. hypogaea* subsp. *fastigiata*
Zenome	AA	AA	BB	BB	AABB	AABB	AABB	AABB
Biological status	Wild	Wild	Wild	Wild	Wild	Cultivated	Cultivated	Cultivated
Accessions	PI475845	V14167	K30076	ICG 8206	PI 263393	cv. Tifrunner	var. Shitouqi	Fuhuasheng
Sequencing platform	Illumina HiSeq2500	Illumina HiSeq2000	Illumina HiSeq2000	Illumina HiSeq2500	Pacbio, BioNano optics and Hi-C	PacBio and Illumina HiSeq 2500	PacBio and Illumina HiSeq 2000	Illumina HiSeq and PacBio
Genome size	1.38 Gb	1.211 Gb	1.512 Gb	1.39 Gb	2.62 Gb	2.552 Gb	2.54 Gb	2.55 Gb
Number of scaffolds	8173	635,392	759,499	79,408	3417	384	1297	86
N50 scaffolds (Kb)	649.84	947.95	5,343.28	170.05	124.92 Mb	9.0 Mb	135.11 Mb	56.57 Mb
GC content (%)	31.79	34.00	35.49	36.70	35.99	36.21	36.53	36.33
Number of gene models	50,324	36,734	41,840	39,704	43,961	66,469	83,709	83,087
Mean number of exon per gene	3.37	NA	NA	4.99	NA	NA	6.82	3.83
Mean exon length (bp)	312	NA	NA	250.0	NA	NA	233.21	278.23
Mean intron length (bp)	709	NA	NA	625.0	NA	NA	599.66	646.32
Number of miRNA gene	801	NA	NA	71.0	NA	NA	480	241
Number of rRNA genes	115	NA	NA	313.0	NA	NA	3,107	3,511
Number of tRNA genes	913	NA	NA	2,914.0	NA	NA	4,723	2,239
Small nuclear RNAs (snRNAs)	202	NA	NA	152.0	NA	NA	30,817	25,299
Transposable elements	59.77%	61.73%	68.50%	75.97%	NA	74.03%	69.23%	54.34%
References	Chen et al. (2016)	Bertioli et al. (2016)		Lu et al. (2018)	Yin et al. (2018)	Bertioli et al. (2019)	Zhuang et al. (2019)	Chen et al. (2019)

The year 2019 has been very fruitful for the entire groundnut research community as three independent efforts made available two reference genomes for subsp. *fastigiata* (Chen et al. 2019; Zhuang et al. 2019) and one for subsp. *hypogaea* (Bertioli et al. 2019). The IPGI-led initiative completed sequencing of *A. hypogaea* subsp. *hypogaea* ‘Tifrunner’ (PI 644,011; ~ 2.56 Gb; 20 pseudomolecules; 66,469 genes) (https://peanutbase.org/peanut_genome; Bertioli et al. 2019) by deploying several modern sequencing and assembly technologies such as PacBio and Hi-C data/technology. Similar advanced technologies were deployed by two independent efforts in China leading to development of high-quality reference genome assemblies for *A. hypogaea* subsp. *fastigiata* ‘Shitouqi’ with ~ 2.54 Gb genome size and 83,709 genes across 20 pseudomolecules (Zhuang et al. 2019) and ‘Fuhuasheng’ with ~ 2.55 Gb genome size and 83,087 genes across 20 pseudomolecules (Chen et al. 2019). The variety ‘Shitouqi’ is well known Chinese landrace, while the genotype ‘Fuhuasheng’ is a landrace from North China. All these reference genomes now provide an array of opportunities for conducting precise studies for understanding the structure and function of the genome and genes that control key agronomic, stress (biotic and abiotic), yield, nutritional and quality features of the cultivated groundnut.

### Resequencing and genome-wide genetic markers

In addition to improving understanding on genome architecture and gene function, genome sequencing provides access to large number of genome-wide simple sequence repeat (SSR) sites which can play a critical role in genetics and breeding applications. The IPGI-led diploid genomes of *A. duranensis* (A genome) and *A. ipaensis* (B genome) were mined for SSRs leading to identification of 135,529 and 199,957 SSRs, respectively (Zhao et al. 2017). Further comparative diploid genome analysis with each other also identified 515,223 InDels, i.e., 269,973 insertions and 245,250 deletions by comparing *A. duranensis* with *A. ipaënsis* (Vishwakarma et al. 2017b). The genome sequence can also be used as reference genome for single nucleotide polymorphism (SNP) calling which are the most abundant structural variation across the genome. The SNPs serve as genetic markers for conducting high-resolution trait mapping studies and can be achieved by generating sequencing data on diverse/segregating lines and aligning to the reference genomes. The sequencing can either be performed at high coverage (whole-genome resequencing; WGRS), mid-coverage (skim sequencing) or low coverage (genotyping-by-sequencing or RADseq). Recently, the high-coverage WGRS data have been generated for 41 diverse genotypes including 30 tetraploids and 11 diploid accessions leading to discovery of 98,375 SNPs in A subgenome and 65,407 SNPs in B subgenome (Pandey et al. 2017a; Clevenger et al. 2017). Most importantly, 58,233 high informative genome-wide SNPs were used for developing the first high-density genotyping assay, ‘Arachis_Axiom’ SNP array. Such high-density genotyping array have been now routinely used for diversity and trait mapping applications in groundnut.

Low-cost sequencing technologies have made sequencing cost-effective for even larger genome sizes, including groundnut and, therefore, provide a great encouragement for sequencing large diversity and germplasm panels in groundnut. For example, resequencing of 54 diverse accessions (18 wild species, 30 tetraploid groundnut cultivars and 4 synthetic tetraploids) has clarified the cultivated groundnut domestication events after polyploidy and the genetic variation in Chinese breeding experiments (Zhuang et al. 2019). Further, resequencing has been completed for 300 diverse accessions of ICRISAT groundnut reference set (see Varshney et al. 2019) for studying genome-wide structural variations, diversity and genome-wide association study (GWAS). Similar efforts are underway for diverse panels in USA and China. Further reduction in the cost of sequencing would witness the sequencing of entire gene bank collections of groundnut across the globe which will serve as the foundation/reference database of sequences for use in conservation, characterization, trait mapping and trait mobilization in addition to development of cost-effective low–mid–high-density genotyping assays.

### Transcriptome atlases

While the NGS has revolutionized genome sequencing and assembly preparation for new genomes, it also strengthens the improvement of genome annotation through the RNA sequencing (RNA-seq) approach. This approach can improve the genome annotation especially for the genes which encodes for proteins and non-coding RNAs. In eukaryotes, the annotation of genes becomes more difficult with genome complexity as only a very small part of genome encodes for the proteins, for example, only 1.3% of the genome in case of human (Salzberg 2019), and in polyploid plants (including groundnut), accurate annotation is still a challenge. To elucidate this, researchers have started working on the gene atlas that comprises cataloging of genes from a broad range of tissues using RNA-seq data. For groundnut, a gene expression atlas for the subsp. *hypogaea* was developed at the University of Georgia (UGA), USA (Clevenger et al. 2016). This study used 22 different tissues of groundnut representing all critical organs (full development stages of tetraploid Tifrunner cultivar) for generation of gene expression data and identified 8816 putative homeologous genes and spotted over 9000 alterative splicing events and over 6000 non-coding RNAs. Another transcriptome map of groundnut was also created in China by RNA-seq of 39 samples from different tissues and circumstances including biotic and abiotic stresses and hormones treatments, which constitute 91.73% of total annotated genes (https://peanutgr.fafu.edu.cn; Zhuang et al. 2019), i.e., all expressed genes in groundnut were supported by RNA-seq data. Most recently, a gene atlas for subsp. *fastigiata* has been developed using tetraploid genome of same subspecies by analyzing important tissues collected from a drought-tolerant bunch-type varieties (ICGV 91114) (Sinha et al. 2020). Further, the improvement of genome annotation involves integration of proteome and metabolome atlases/map. This information is essential to improve the existing annotation of genomes still containing higher numbers of RNA genes than protein coding ones. In humans, the proteome and the metabolome atlases/map have been recently created (Kim et al. 2014; Uhlén et al. 2015), which has been replicated currently in several crop plants including groundnut. The deeper understanding from genome to metabolome and epigenome will provide clear progression of trait development and expression in crop plants.

### High-, mid-, and low- density genotyping assays

Genotyping assays with different marker density are required to achieve cost-effectiveness in diverse genetics and breeding applications. SNPs are now the most preferred genomic variations to be used as genetic markers due to their abundance in plant genomes and also their amenability for low-cost high-throughput genotyping. The low-density assay may have 10–100 polymorphic SNPs and can be used as quality control (QC) for purity testing of founder parents and its progenies in breeding programs. The mid-density assay with 2000–5000 SNPs is very much required for use in genomic selection, while high-density assay with > 10 K SNPs is optimum for performing genetic diversity, genome-wide association and genetic mapping studies. In case of groundnut, the community has access to only high-density genotyping platform ‘Axiom*_Arachis*’ SNP arrays which have 58,233 highly informative genome-wide SNPs and was developed after sequencing 41 diverse genotypes (30 tetraploids and 11 diploids) (Pandey et al. 2017a). The high-density genotyping array has shown preference over the genotyping-by-sequencing (GBS) as the latter gives a large proportion of missing data which needs imputing further to complete the genetic analysis. The deployment of this high-density SNP assay showed significant diversity loss in cultivated gene pool and preferential selection for different subspecies (Pandey et al. 2017a) in addition to discovery of positive selection in genomic regions during the course of breeding of US runner-type breeding material (Clevenger et al. 2017). Therefore, the above developed high-density genotyping assay is playing an important role in performing high-resolution genetic diversity, trait mapping and genetic background recovery studies in groundnut. Among sequencing-based genotyping approaches, the whole-genome resequencing (WGRS) and skim sequencing provide high-density mapping opportunity, while the GBS, restriction site-associated sequencing (RADSeq), double-digest restriction site-associated sequencing (ddRADseq) and specific length amplified fragment sequencing (SLAF-seq) technologies provide mid-density genotyping technology. In coming years, cost-effective low-density and mid-density genotyping assays will be developed and deployed for different genetic and breeding applications in groundnut. In fact, the mid-density genotyping assay should also be enriched with trait-linked diagnostic markers to broaden its application for performing early generation selection and background selection in addition to its deployment in routine genomic selection breeding program.

### High-throughput and cost-effective genotyping assay with diagnostic markers for early generation selection

Marker-assisted selection (MAS) and marker-assisted backcrossing (MABC) are the two most successful molecular breeding approaches which have been deployed successfully in several crops species. It is now routine in many crop species to deploying trait-linked markers in breeding including groundnut. All the trait mapping studies should not only be directed for identifying associated genomic regions with traits, as it is happening in most cases; in fact, the most important aspect is gene discovery followed by development and validation of diagnostic markers. However, achieving diagnostic markers is not an easy task due to several factors having large impact such as precise phenotyping and dense genotyping on the genetic populations. Nevertheless, the sequence-based trait mapping approaches are taking researchers not just to smaller genomic regions but also providing access to the candidate genes and markers (Pandey et al. 2016; Varshney et al. 2019; Zhuang et al. 2019). In case of groundnut, the diagnostic SNP markers have been developed for high oleic acid (Chu et al. 2009), root-knot nematode resistance (Chu et al. 2011), foliar disease resistance (Pandey et al. 2017b), leaf spot and tomato spotted wilt virus resistance (Agarwal et al. 2018, 2019), seed size (Zhuang et al. 2019), shelling percentage (Luo et al. 2019a) and bacterial wilt resistance (Luo et al. 2019b). The validated SNPs are now available for developing smaller SNP panels for making early generation selection in breeding. ICRISAT is leading High Through-Put Genotyping (HTPG, https://cegsb.icrisat.org/high-throughput-genotyping-project-htpg/) project funded by the Bill & Melinda Gates Foundation. This project facilitates genotyping of 10 SNPs for just US$ 1.5 per sample including DNA extraction. To avail this opportunity, ICRISAT has developed and deployed a 10-SNP panel containing diagnostic SNP markers for resistance to rust and late leaf spot as well as high oleic acid (Pandey et al. 2019c). So far, this 10-SNP panel has been used to genotype more than 55,000 breeding lines for performing early generation screening in India, Malawi, Mali, Niger, Uganda, Senegal and Ghana. In coming years, more such panels for different traits will become available and the cost may further reduce leading to wider adoption in routine breeding program across the globe.

## Shift from conventional to sequence-based faster discovery of genomic regions and gene discovery

Technological advancements in sequencing and high-throughput genotyping have provided great acceleration to the trait discovery efforts in addition to providing opportunities to perform high-resolution mapping and faster candidate gene discovery. Huge reduction in sequencing cost and availability of high-quality reference genomes in groundnut have made sequence-based trait mapping more affordable and time efficient, which is reflected in publications of several such studies in groundnut during last 2–3 years (Table 2). Nevertheless, all such studies conducted until now used diploid genomes; however, from now on, new studies will be performed using tetraploid genomes which have become available now for both the subspecies of cultivated groundnut. In a broader sense, currently the sequencing-based mapping is being performed either by sequencing complete populations or by sequencing pooled samples with extreme phenotypes of the target trait (Pandey et al. 2016; Varshney et al. 2019). The low coverage sequencing approaches include GBS (Elshire et al. 2011; Poland et al. 2012), RADseq (Miller et al. 2007), ddRADseq (Peterson et al. 2012) and SLAF-seq (Sun et al. 2013), and high coverage sequencing approach, namely WGRS, was used in groundnut for getting large-scale genome-wide SNPs in mapping populations and developing high-density genetic maps for conducting high-resolution trait mapping.

**Table 2:** Summary of sequencing-based trait mapping efforts in cultivated groundnut

Sequencing strategy/ platform	Mapping population	Trait mapping approach	Target traits	Significant outcome	Reference
ddRADseq	Zhonghua 5 × ICGV 86699	Genetic mapping (1621 SNP loci)	LLS resistance and plant-type-related traits	Identified small-effect QTLs for LLS and other traits	Zhou et al. (2014 )
ddRADseq	Xuhua 13 × Zhonghua 6	Genetic mapping (2,595 SNP loci)	Oil content	Seven QTLs for oil content including the major and stable QTL qOCA08.1 with 10.14–27.19% PVE	Liu et al. (2020)
RADSeq	TAG 24 × GPBD 4	Genetic mapping (171 SNP loci)	Rust and LLS resistance	Identified QTLs for LLS and rust resistance on 1.4- and 2.7-Mb genome regions on chromosomes A02 and A03, respectively	Shirasawa et al. (2018)
RADSeq	99 accessions of Chinese mini core collection	GWAS (36,058 SNPs)	Aflatoxin production	60 SNP markers associated with aflatoxin production detected explaining 16.87%–31.70% PVE, with SNP02686 and SNP19994 possessing 31.70% and 28.91% PVE, respectively	Yu et al. (2020)
GBS	Florida-07 × GP-NC WS 16	Genetic mapping (2,753 SNP loci)	ELS and LLS resistance	Identified major QTL for LLS resistance anchored by two NBS-LRR resistance genes on chromosome B05. Two major QTLs for ELS resistance were identified on A03 and B04	Han et al. (2018)
GBS	TG37A × NRCG-CS85	Genetic mapping (585 SNP loci)	Stem rot resistance	Identified 44 major epistatic QTLs with 14.32 to 67.95% PVE on B04 harboring 170 resistance genes such as LRR, ERF and zinc finger motifs	Dodia et al. (2019)
GBS	195 groundnut accessions	GWAS (13,435 SNPs)	Yield-related traits	Gene annotation for 12 co-localized SNP detected 36 candidates genes, and one of these gene (*arahy. RI9HIF*) hold the potential for significantly improving peanut yield	Wang et al. (2019)
GBS	TAG 24 × GPBD 4	Genetic mapping (585 to 2753 SNP loci)	Rust and LLS resistance	Identified one major QTL for rust resistance (A03) and two major QTLs (A03 and A02) for LLS resistance. These QTL regions harbor > 200 candidate gens and six of these genes showed direct or indirect involvement in biotic stress with deleterious InDels/SNPs	Pandey et al. (2017c)
SLAF-seq	Huayu28 × P76	Genetic mapping (2,266 SNP loci)	Oil quality traits	First high-density genetic map based on SLAF and identified associated genomic regions and markers for oleic and other fatty acids	Hu et al. (2018)
SLAF-Seq	Jihua 5 × M130	Genetic mapping (2,808 SNP loci)	Growth habit-related traits	39 QTLs were detected for growth habit-related traits with 4.55– 27.74% PVE; 6 QTLs for lateral branch angle, 8 QTLs for extent radius, 7 QTLs for the index of plant type, 11 QTLs for main stem height, and 7 QTLs for lateral branch length	Li et al. (2019)
SLAF-seq	Huayu36 × 6–13	Genetic mapping (3,866 SNP loci)	Seed weight and size	Two stable QTL regions identified on chromosomes 2 and 16, and gene content provided valuable information for further functional analysis of yield component traits	Zhang et al. (2019)
SLAF-seq	ZH16 × sd-H1	Genetic mapping (3,630 SNP loci)	Yield related traits	Two stable co-located QTLs for seed- and pod-related traits were significantly identified in the chromosomal end of B06 and B07, respectively	Wang et al. (2018)
RNA-Seq	Zhonghua 10 × ICG 12,625	eQTLs	Peanut purple testa color	Unveiled a putative candidate gene and developed a linked InDel marker for purple testa color	Huang et al. (2020)
WGRS	Tifrunner × GT-C20	Genetic mapping (8869 SNP loci)	ELS, LLS, and TSWV resistance	Identified two QTLs for ELS on B05 with 47.42% PVE and B03 with 47.38% PVE, and two QTLs for LLS on A05 with 47.63% and B03 with 34.03% PVE and one QTL for TSWV on B09 with 40.71% PVE	Agarwal et al. (2018)
WGRS	SunOleic 97R × NC94022	Genetic mapping (11,106 SNP loci)	TSWV resistance	Identified three QTLs co-localized on chromosome A01 and one of these QTL showed 36.51% PVE for TSWV resistance limited to 89.5 Kb genomic region. Cluster of resistance genes identified and KASP markers developed and validated	Agarwal et al. (2019)
WGRS	TAG 24 × GPBD 4	QTL-seq (3,136 SNP loci)	LLS resistance	Identified genomic region on A03 that explains > 80% PVE for rust and > 40% PVE for LLS resistance. Identified 19 candidate genes and validated 6 markers for rust and LLS resistance. Developed and validated low-cost KASP genotyping assay	Pandey et al. (2017b)
WGRS	Florida-07 × GP-NC WS 16	QTL-seq (5,513 SNP loci)	LLS resistance	Identified significant candidate QTLs on chromosomes, A05, B03, and B05; and developed three KASP markers explaining 15% PVE for LLS resistance	Clevenger et al. (2018)
WGRS	Xuhua 13 × Zhonghua 6	QTL-seq (358 SNP loci)	Bacterial wilt resistance	Identified one major QTL, qBWRB02.1 on B02 with 13.02%–40.19% PVE. Cost-effective KASP assay developed and validated	Luo et al. (2019a)
WGRS	Yuanza 9102 × Xuzhou 68–4	QTL-seq (455 SNP loci)	Shelling percentage (SP)	Identified two overlapped genomic regions (2.75 Mb on A09 and 1.1 Mb on B02) harboring 9 candidate genes. Cost-effective KASP assay developed and validated	Luo et al. (2019b)
WGRS	YH29 × ZH9 and WH10 × ZH8	QTL-seq and BSR-Seq (1797 SNP loci)	Peanut purple testa color	The purple testa color is controlled by female parent and identified single major gene, AhTc1 encoding a R2R3-MYB transcription factor, followed by successful development of allele-specific markers	Zhao et al. (2020)
WGRS	ICGV 00350 × ICGV 97045	QTL-seq (10759 SNP loci)	Fresh seed dormancy	Two candidate genes—*RING-H2* finger protein and *zeaxanthin epoxidase* were identified, which significantly express during seed development and control abscisic acid (ABA) accumulation	Kumar et al. (2020)

*ddRADseq* double-digest restriction-site-associated DNA sequencing,*RADseq* restriction site-associated sequencing, *GBS* genotyping-by-sequencing, *SLAF-seq* specific length amplified fragment sequencing, *WGRS* whole-genome resequencing, *eQTLs* expression quantitative trait loci, *KASP* Kompetitive allele-specific PCR, *TSWV* tomato spotted wilt virus

The RADseq technology was initially checked for level of polymorphism among a set of parental genotypes of genetic populations (Gupta et al. 2015); however, it yielded a low-density genetic map (171 SNP loci) when deployed in a RIL population segregating for rust and late leaf spot (LLS) resistance in TAG 24 × GPBD 4 (Shirasawa et al. 2018). Nevertheless, the improved technology named ddRADseq provided much better results when deployed for construction of dense genetic maps (1,621 SNP loci) in RIL population derived from Zhonghua 5 and ICGV86699 (Zhou et al. 2014). The GBS-based sequencing approach was utilized for developing three dense genetic maps (585 to 2753 SNP loci) and successful discovery of genomic regions and candidate genes for resistance to rust and LLS in TAG 24 × GPBD 4 (Pandey et al. 2017c), stem rot in TG37A × NRCG-CS85 (Dodia et al. 2019) and early leaf spot (ELS) and LLS in Florida-07 × GP-NC WS 16 (Han et al. 2018). Deployment of another technology, SLAF-seq, helped in developing denser genetic maps with 2266 SNP loci in RIL population (Huayu28 × P76) and with 2808 SNP loci in RIL population (Jihua 5 × M130), respectively, leading to high-resolution mapping of oil quality traits (Hu et al. 2018) and growth habit-related traits within 0.17 Mb with putative genes (Li et al. 2019). This technology was also successfully used in developing an ultra-higher dense genetic map with 7184 high-quality SNP loci in a RIL population (Yueyou 92 × Xinhuixiaoli) followed by discovery of narrow co-localized regions of several seed size traits leading to isolation of key candidate genes. Candidate genes for testa color were also discovered using the same dense map for fine trait mapping (Zhuang et al. 2019). The SLAF-seq technology on a diverse panel with 158 accessions identified 17,338 high-quality polymorphic SNPs which were then used for conducting population structure, linkage disequilibrium (LD) decay and genome-wide association study (GWAS) for major agronomic traits related to domestication (Zhang et al. 2017). In order to achieve a very high number of polymorphic SNPs, WGRS of RIL population (Tifrunner × GT-C20) facilitated development of high-density genetic maps (8869 to 11106 SNP loci) leading to fine mapping and candidate gene discovery for resistance to ELS, LLS and TSWV (Agarwal et al. 2018, 2019). With the exception of one study (Li et al. 2019) using the recently available tetraploid genome, the SNP calling has been performed on diploid reference genomes (Bertioli et al. 2016) for all other previous studies. The availability of quality reference genomes for both the subspecies of cultivated groundnut(Bertioli et al. 2019; Chen et al. 2019; Zhuang et al. 2019) will further enhance the precision and accuracy of sequence alignment and SNP calling which will eventually facilitate precise discovery of genomic regions and candidate genes (Zhuang et al. 2019).

Among all the pooled sequencing-based approaches, namely QTL-Seq, MutMap, Seq-BSA InDel-Seq and BSR-Seq (see Pandey et al. 2016), the ‘QTL-Seq’ has been successfully applied for discovery of genomic regions and candidate genes in groundnut (Pandey et al. 2017b; Clevenger et al. 2018; Luo et al. 2019a, b; Zhuang et al. 2019; Kumar et al. 2020; Zhao et al. 2020). The first QTL-seq study using diploid reference genomes identified a co-localized genomic region on chromosome A03 for rust and LLS resistance in TAG 24 × GPBD 4 followed by development and validation of allele-specific diagnostic markers for rust and LLS resistance (Pandey et al. 2017b). The same dataset was then used against the tetraploid genome (Zhuang et al. 2019), with the same co-localized genomic region being detected in chromosome 13 (B03) providing a perfect example of translocation (Chr03 to Chr13) after tetraploidization. The other such study on LLS resistance in Florida-07 × GP-NC WS 16 population identified multiple genomic regions which were validated by QTL mapping, backward and blind selection, and the selection with associated markers showed significant increase in resistance in the field (Clevenger et al. 2018). The third QTL-seq study was performed for shelling percentage in the above-mentioned population (Yuanza 9102 × Xuzhou 68–4) identified two genomic regions (2.75 Mb on A09 and 1.1 Mb on B02) and 9 candidate genes followed by development of cost-effective KASP (Kompetitive Allele-Specific PCR) assay for use in breeding (Luo et al. 2019a). The fourth QTL-seq analysis for bacterial wilt resistance in Xuhua 13 × Zhonghua 6 identified one candidate genomic region and candidate genes on chromosome B02 followed by development and validation of two diagnostic markers for use in breeding (Luo et al. 2019b). The fifth QTL-seq study was performed for seed size (one bulk with 54 big seeded RILs and second bulk with 54 small seeded RILs) in the population (Yueyou92 × Xinhuixiaoli) getting two QTL-conformed regions leading to isolation of candidate genes between two co-segregated SNPs (Zhuang et al. 2019). The sixth QTL-Seq study determined that the purple testa color is controlled by female parent and identified a single major gene, AhTc1 encoding a R2R3-MYB transcription factor, followed by successful development of allele-specific markers (Zhao et al. 2020). Most recently, the seventh QTL-seq analysis in RIL population (ICGV 00350 × ICGV 97045) for fresh seed dormancy identified two candidate genes—*RING-H2 finger protein* and *zeaxanthin epoxidase* which significantly express during seed development and control abscisic acid (ABA) accumulation (Kumar et al. 2020). More such studies are likely to be conducted for different traits in groundnut in coming years.

All the above sequencing-based trait mapping were performed on the complete biparental population using the DNA samples. The lone study has come recently in groundnut which deployed RNA-Seq analysis by sequencing RNA samples from complete RIL population (Zhonghua10 × ICG12625) (Huang et al. 2020). This study reported 49,691 genes, and 92 of these genes were found to follow paramutation-like expression pattern. The expression quantitative trait loci (eQTL) analysis reported 1207 local eQTLs and 15,837 distant eQTLs followed development of linked marker InDel02 for purple testa color in groundnut. As far as using RNA-Seq analysis on pooled samples is concerned, the Zhao et al. (2020) performed bulked segregant RNA sequencing (BSR-Seq) for purple testa color and confirmed the results achieved through QTL-Seq analysis by achieving similar results. It is worth mentioning that the BSR-Seq approach provides a cheaper option for candidate gene discovery as compared to QTL-Seq approach; nevertheless, the QTL-Seq generated more SNPs across the genome than BSR-Seq which helps in narrowing down the candidate genomic regions and genes (Zhao et al. 2020).

These sequencing-based trait mapping efforts not only identified genomic regions and candidate genes but also facilitated development and validation of diagnostic markers which are being used currently in routine breeding programs.

## Genome to field: success stories on translating genomic information for developing molecular breeding products

In the last decade, the groundnut research community has made extraordinary efforts in developing optimal genomic resources that are essential for translating genomic information for use in crop improvement programs. Groundnut has now attained the status of crop with optimum genomic resources which will facilitate faster discovery of genes and linked markers for use in genomics-assisted breeding (GAB). Even with limited genomic resources, the diagnostic markers have successfully been developed in groundnut for high oleic acid, and resistance to nematode, rust and LLS. Of the three GAB approaches, namely marker-assisted selection (MAS) or marker-assisted backcrossing (MABC), marker-assisted recurrent selection (MARS) and genomic selection (GS), the MAS/MABC has been the most successful approaches, while the optimization of GS is still in its initiation phase in groundnut. We have summarized the GAB in groundnut under following three subheads, i.e., MAS/MABC efforts, GS initiative and molecular breeding products in farmers’ field (Table 3).

**Table 3:** Summary of successful molecular breeding efforts in groundnut

S. No	Trait	Donor parent	Recurrent parent	Current status/ Variety released	References
1	High oleic acid and root-knot nematode resistance	Tifguard, Georgia-02C and Florida-07	Tifguard	Pyramided nematode resistance and the high oleic acid through MAS followed by 3 backcrosses with Tifguard to develop ‘Tifguard High O/L’ variety. First molecular breeding product released as variety for commercial cultivation in USA	Chu et al. (2011)
2	Rust resistance	GPBD 4	TAG 24, JL 24 and ICGV 91114	Developed hundreds of backcross lines followed by multi-location yield trials. These molecular breeding lines (ICGV 14421, ICGV 13189 and ICGV 13207) are in final year of testing for release in India	Varshney et al. (2014), Janila et al. (2016a)
3	High oleic acid	SunOleic 95R	ICGV 06110, ICGV 06142 and ICGV 06420	Developed hundreds of high oleic backcross lines followed by multi-location yield trials in India. Two of these high oleic molecular breeding lines are released as ‘Girnar 4 (ICGV 15083)’ and ‘Girnar 5 (ICGV 15090)’ for commercial cultivation in India	Janila et al. (2016a, b)
4	Rust and late leaf spot (LLS) resistance	GPBD 4	JL24	Developed several backcross lines, namely JG4_81, JG4_43, JG2–3_14, JG_18, which are currently under field evaluation	Yeri and Bhat (2016)
5	Rust and late leaf spot resistance	GPBD 4	TMV 2	Developed two promising molecular breeding lines, namely TMG 29 and TMG 46, which are currently under field evaluation	Kolekar et al. (2017)
6	High oleic acid	SunOleic 95R	ICGV 05141	Developed several molecular breeding lines and identified best promising line, namely ICGV 05141, based on multiplication trials	Bera et al. (2018)
7	High oleic acid and resistance to rust and LLS	SunOleic 95R	GPBD 4	Improved a highly resistant variety, GPBD 4 for high oleic acid. Further yield trials under way	Nawade et al. (2019)
8	High oleic acid	SunOleic 95R	ICGV 06100	Several high oleic molecular breeding lines developed in the genetic background of ICGV 06100 which are currently under yield trials	Bera et al. (2019)
9	High oleic acid	Kainong176, DF12, Kainong176 and KX01-6	Yuhua 15, Yuanza 9102, ‘Yuhua 9326, and Yuhua 9327	The promising and superior lines selected now available for conducting multi-location yield trials for cultivar registration and release	Huang et al. (2019)
10	High oleic acid, resistance to rust and LLS	SunOleic 95R and GPBD 4	GJG 9, GG 20 and GJGHPS 1	> 50 FDR MABC lines and > 80 high oleic lines in BC_3_F_7_ generation were developed which are being now subjected to seed multiplication	Shasidhar et al. (2020)
11	High oleic acid, resistance to rust and LLS	SunOleic 95R and GPBD 4	Dh86, ICGV 87846, ICGV 00351 and Kadiri 6	Several MABC and pyramided lines developed and are now available for further evaluation, testing and release	ICRISAT unpublished

### Marker-assisted improvement of popular US cultivar for nematode resistance and high oleic acid at University of Georgia, USA

The first attempt was made at University of Georgia (UGA), USA, by using linked markers for nematode resistance and high oleic acid to improve nematode-resistant cultivar ‘Tifguard’ for high oleic acid through two parallel backcrossing programs using two donor high oleic parents, namely Georgia-02C and Florida-07 (Chu et al. 2011). The BC_3_F_2_ plant progenies were confirmed for homozygosity of target alleles (nematode resistance and high oleic acid) and phenotypes followed by generation advancement and yield trials.

### Marker-assisted improvement of three Indian popular varieties for resistance to rust and LLS at ICRISAT, India

The second attempt was made at ICRISAT, India, for improving resistance to rust and LLS by deploying SSR markers in three Indian popular varieties, namely ICGV 91114, JL 24 and TAG 24 (Varshney et al. 2014). The backcrossed homozygous lines (BC_3_F_2_) were obtained in just three years of time which were further generation advanced after trait confirmation and were then subjected to multi-location yield trials together with national partners in India. Some of these MABC lines (ICGV 13229, ICGV 13192, ICGV 13193, ICGV 13206, ICGV 13200 and ICGV 13228) showed significant increase in pod yield (56–96%) and haulm yield (25–89%) in addition to breaking the much needed genetic linkage between foliar disease resistance and long maturity duration (Janila et al. 2016a). As a result in the genetic background of TAG 24, ICGV 91114 and JL 24, a total of 12 MABC lines (ICGVs 13189, 13193, 13202, 13207, 13219, 13220, 13221, 13229, 14410, 14415, 14421 and 14431) were nominated to All India Coordinated Research Project on Groundnut (AICRP-G) of Indian Council of Agricultural Research (ICAR), which is the nodal agency for evaluation and recommendation for release of variety in India for cultivation.

### Marker-assisted improvement of three Indian popular varieties for high oleic acid at ICRISAT, India

The third MABC and MAS effort was successfully implemented at ICRISAT by enhancing the high oleic level in three Indian popular Spanish and Virginia groundnut varieties, namely ICGV 06420, ICGV 06110 and ICGV 06142 (Janila et al. 2016b). The allele-specific markers for *FAD2A* and *FAD2B* genes were deployed for selecting mutant alleles in the segregating breeding populations. Here, emphasis was given on developing improved lines with two combinations, i.e., 27 high oil content (53–58%) + high oleic acid (~ 80%) lines to meet the demand of making available quality cooking oil and 28 low oil content (42–50%) + high oleic acid (~ 80%) lines for confectionary/other food uses. As a result, several molecular breeding lines with ~ 80% oleic acid were developed by increasing from ~ 60% oleic acid in recurrent parent to ~ 80% oleic acid in molecular breeding lines. Preliminary yield and quality data were then generated for these molecular breeding lines at ICAR-Directorate of Groundnut Research (DGR, Junagadh, Gujarat), Junagadh Agricultural University (JAU, Junagadh, Gujarat) and Tamil Nadu Agricultural University (TNAU, Coimbatore, Tamil Nadu). From these, two molecular breeding lines (ICGV 15105 and ICGV 15106) in the genetic background of recurrent parent ICGV 06142 and 14 molecular breeding lines (ICGV 15006, ICGV 15016, ICGV 15017, ICGV 15035, ICGV 15052, ICGV 15064, ICGV 15065, ICGV 15070, ICGV 15073, ICGV 15074, ICGV 15080, ICGV 15083, ICGV 15090 and ICGV 15095) in the genetic background of recurrent parent ICGV 06420 were nominated by different Indian collaborators to AICRP-Groundnut for testing and release. After two years of testing in six locations (Junagadh–Gujarat, Durgapura-Rajasthan, Dharwad–Karnataka, Tindivanam–Tamil Nadu, Tirupati–Andhra Pradesh and Palem–Telangana) in India during rainy 2017 and 2018, two best performing entries nominated by the ICAR-DGR, Junagadh, ICGV 15083 and ICGV 15090, have been identified in April 2019 by the Varietal Identification Committee of ICAR to be released as varieties, Girnar 4 (ICGV 15083) and Girnar 5 (ICGV 15090) by ICAR-DGR, Junagadh for cultivation in six major groundnut growing states of India, namely Gujarat, Rajasthan, Karnataka, Tamil Nadu, Andhra Pradesh and Telangana.

### Marker-assisted improvement of popular varieties JL 24 and TMV 2 for resistance to rust and LLS at University of Agricultural Sciences (UAS), Dharwad, India

The fourth MABC effort successfully improved the resistance in an Indian popular variety, JL 24, for two foliar fungal diseases using linked markers at University of Agricultural Sciences (UAS-Dharwad), Dharwad, India (Yeri and Bhat 2016). Evaluation of backcross lines successfully identified several promising lines, namely JG4_81, JG4_43, JG2–3_14, JG_18, which have shown to have equivalent performance for yield and disease resistance with the recurrent parent, JL 24. At UAS-Dharwad, the fifth MABC effort improved the TMV 2 variety, which is a very popular groundnut variety among the Indian farmers but is highly susceptible to LLS and rust (Kolekar et al. 2017). Finally, two homozygous backcross lines, namely TMG‐29 and TMG‐46, were identified which showed resistance to rust and LLS (score of 3.0 in 9.0 scale) in addition to 62.7–71.0% increase in pod yield over the recurrent parent, TMV 2 under the station trials. The backcross lines in the genetic background of JL 24 and TMV 2 have successfully completed the multi-location trials showing ~ 15% yield advantage over their recurrent parents. Now, the large-scale demonstration and seed multiplication have been undertaken for these lines to conduct farm trials followed by their nomination to AICRP-G, India, for further testing and release.

### Marker-assisted improvement of three Indian popular varieties for high oleic acid at ICAR-Directorate of Groundnut Research (DGR), Junagadh, India

The sixth successful MABC effort improved oil quality by altering levels of fatty acid and increasing oleic to acid up to ~ 80% in the Indian popular variety, ICGV 05141, a high oil content genotype selected based on multiplication trials (Bera et al. 2018). ICAR-DGR also targeted FDR variety, GPBD 4, and improved its oleic acid to 80% using MABC approach (seventh effort) by deploying allele-specific and CAPS markers (Nawade et al. 2019). The high yielding variety, GPBD 4, had already natural variation for *FAD2A* mutant allele, therefore, foreground selection was only made for *FAD2B* mutant allele. In addition to the above variety, ICAR-DGR also made the eighth effort and successfully increased oleic acid in another high oil content containing variety, ICGV 06100, by introgressing mutant alleles using MABC approach (Bera et al. 2019). Phenotyping of molecular breeding lines grown in different locations showed stable expression for all the three major fatty acids, namely oleic acid, linoleic acid and palmitic acid. Several high oleic molecular breeding lines are now available in the genetic background of ICGV 05141, GPBD 4 and ICGV 06100 which have shown equivalent performance with the recurrent parent in terms of important agronomic features such as pod yield and shelling percentage.

### Marker-assisted improvement of four Chinese popular varieties for high oleic acid at Henan Academy of Agricultural Sciences, Zhengzhou, China

The ninth effort came from China where four popular groundnut cultivars (Yuhua 15, Yuanza 9102, Yuhua 9326 and Yuhua 9327), cultivated in large growing areas in China, were successfully improved for high oleic acid using the MABC approach (Huang et al. 2019). A total of 24 high-oleic-acid lines (BC_4_F_4_ and BC_4_F_5_) possessing similar morphological and agronomic traits similar to recurrent parents were developed within 5 years. This study also used the KASP assay for performing background selection which helped in selection of promising molecular breeding lines having up to 92.31% genome recovery. The promising and superior lines selected are now available for conducting multi-location yield trials for cultivar registration and release. More importantly, this study used a single high-throughput and cost-effective KASP assay with 27 SNPs for performing foreground and background selection in a groundnut breeding program.

### Marker-assisted pyramiding of resistance to rust and LLS and high oleic acid in three Indian varieties at ICRISAT, India

The tenth successful MABC effort was made to improve three popular Indian varieties, namely GJG 9, GG 20 and GJGHPS 1, for foliar disease resistance (FDR) and high oleic acid. As a result, > 50 FDR MABC lines and > 80 high oleic lines in BC_3_F_7_ generation were developed which are now being now subjected to seed multiplication (Shasidhar et al. 2020). More importantly, this study also deployed 58 K Axiom_*Arachis* array and identified molecular breeding lines with up to 94% recurrent parent genome recovery among second and third backcross progenies. Similarly, > 200 MABC lines (BC_3_F_4_) have been generated during the eleventh MABC effort for another set of three Indian popular varieties, namely Dh86, ICGV 87846, ICGV 00351 and Kadiri 6 for high oleic acid and FDR (ICRISAT unpublished). In addition to generation of MABC lines for individual traits, the above backcrossed lines were also used for developing > 200 pyramided lines by combining both these traits in the genetic background of all the six varieties. The above MABC and pyramided lines are now available for further evaluation, testing and release.

### Mutant allele selection leading to new high oleate groundnut

To create new high oleate groundnut, two cultivars, Minhua 6 and Minhua 8, were treated with EMS and gamma-ray, respectively, and their offsprings screened for known *FAD2A* and *FAD2B* sequences based on genome sequences (Zhuang et al. 2019). Four high oleic varieties with more than 80% oleic acid derived from Minhua 6 and three from Minhua 8 were bred with better agronomic traits of yield performance, quality and/or resistance. Two varieties are now being tested in regional trials for potential release. Through the same methods, high yield and quality cultivars, Zhonghua16 and Dihao2 were also mutated by EMS at Fujian Agriculture and Forestry University and a series of high oleate varieties with different characteristics were created for further selection and characterization. As mutagen can cause thousands of SNPs and InDels to a specific variety, many new mutants can be created at the same time which could supply a source for functional genes identification in the post-genomic era. With the large-scale gene editing approaches being developed for groundnut, these methods should create targeted mutations or genetic materials for functional genes studies and meeting human needs in the near future.

## Optimization of GS breeding to improve complex traits

Genomic selection (GS) has emerged as the promising new molecular breeding approach for improving even complex traits in less time and with more precision and accuracy (Meuwissen et al. 2001; Crossa et al. 2017; Xu et al. 2020). In case of GS, development of training and testing populations is a prerequisite for optimizing the genomic prediction of different GS models, wherein the precise multi-location phenotyping data play a key role in addition to genotyping with optimum density required based on the genome size. It is important to mention that GS approach not only promises to handle complex traits but also provides the additional advantage by reducing the selection cycle and avoiding extensive phenotyping through the selection of the superior lines based on prediction of the genomic-estimated breeding values (GEBV) (Crossa et al. 2017). However, the prediction accuracy in GS approach is affected by several factors such as size of the training population and its constitution/structure, precise and quality phenotyping, marker density, and trait heritability (Xu et al. 2020).

**Fig. 4:**
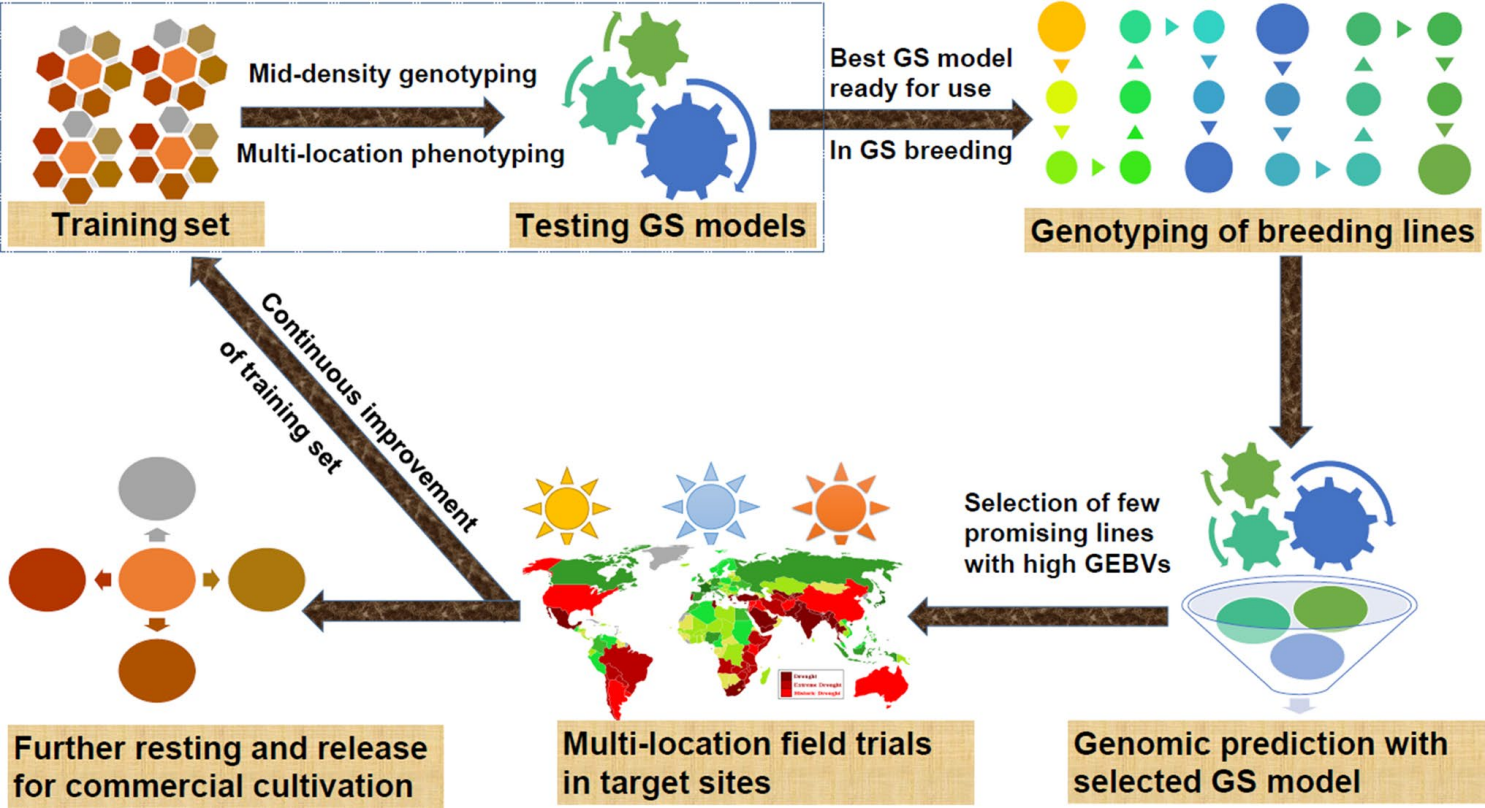
Genomic selection breeding strategy in groundnut. The availability of mid/high-density genotyping assays have provided opportunity of deploying the genomic selection in groundnut. The training population has been developed followed by its multi-season phenotyping data and genotyping with high-density genotyping. Appropriate GS models are being optimized for initiating GS breeding in groundnut

ICRISAT has taken some initiatives together with its NARS partners toward optimizing and deploying GS breeding in groundnut (Fig. 4). Initially, the genotyping data on 2,356 DArT polymorphic markers were analyzed together with six seasons of phenotyping data on ICRISAT minicore collection for days to flowering (DF), seed weight (SW) and pod yield (PY) with varied heritability (Pandey et al. 2014, 2015). The study showed higher prediction accuracies with associated markers as compared to total markers in complete minicore collection; however, the same was not observed when a smaller set of lines from minicore collection were considered as the training population. Irrespective of the size of the training and validation sets, a positive correlation was observed between the high trait heritability and high prediction accuracy. In order to develop much needed high-density genotyping assay for use in GS breeding, 58 K Axiom_*Arachis* SNP array was developed and validated for accelerating the groundnut genetic and genomics research (Pandey et al. 2017a; Clevenger et al. 2017). Later on, ICRISAT worked together with national partners in India and constituted a training population with 340 elite groundnut lines compassing the trait diversity required for Indian groundnut breeding programs. This training population is being phenotyped for 11 agronomic, 7 quality and 6 foliar disease resistance traits at four locations (Patancheru, Aliyarnagar, Jalgaon and Dharwad) in India (Chaudhari et al. 2019). High-density genotyping data have been generated using 58 K SNP assay, and 13,355 genome-wide polymorphic SNPs have been identified for performing GS analysis. More importantly, an Indo-UK dedicated project has been initiated by ICRISAT together with The Roslin Institute, The University of Edinburgh, UK, which targets to genotype and phenotype ~ 10,000 breeding lines followed by optimizing the GS strategy and appropriate model for deployment in groundnut GS breeding for complex traits such as oil content and pod yield.

## Rapid generation advancement and speed breeding

With the ever-increasing global population, the current rate of varietal development and replacement in farmer’s field is still very low. More than often it takes more than a decade to develop improved varieties that are subsequently commercialized in farmers’ field. Therefore, the new technology ‘rapid generation advancement’ or ‘speed breeding’ has come to the rescue by shortening the life cycle of a crop species, and therefore, allowing researchers to make more generations in a year. Although this concept is not new for groundnut (Nigam et al. 1994; O’Çonnor 2012; O’Çonnor et al. 2013), the recent emphasis on it has brought more awareness and realization for this technology (Watson et al. 2018; Hickey et al. 2019).

Studies on effects of temperature and photoperiod on vegetative and reproductive growth in groundnut provided basic information on the possibility of shortening the life cycle under controlled conditions (Nigam et al. 1994). This study performed experiments under controlled‐environment conditions in growth chambers using three temperature regimes (22/18, 26/22 and 30/26 °C, day/night) to assess performance of genotypes under long day (12 h) and short day (9 h) photoperiods and suggested that the pod-to-peg ratio (PPR) could be used as indicator of genotypic sensitivity to assess photoperiod effect in groundnut. The speed breeding technology was then optimized and used in groundnut to make at least one more generation per calendar year to increase generation advancements (O'Connor 2012; O'Connor et al. 2013). These studies initially performed optimized ideal plant population in large pots and examined the impact of 24-h light system to determine genotypic variation on photoperiod sensitivity. One of these studies successfully deployed speed breeding techniques in breeding rust-resistant groundnut lines (O'Connor 2012). Another study by same group implemented a speed breeding system for rapid generation of a population starting from F_2_ to F_5_ generation under controlled greenhouse conditions (O'Connor et al. 2013) showing that four generations/year are possible in groundnut.

Realizing the importance of speed breeding in rapid generation advancement in groundnut, a fresh momentum can be seen to fine tune this technology for using it in different genomics and breeding applications. Today this technology is possible for hundreds of plants, which may transform further to handle hundreds of thousands of plants at one go. Among major applications of speed breeding, the major possible applications in groundnut include (a) faster development of genetic populations such RILs, NAM, MAGIC and NILs for trait mapping, (b) accelerated domestication and faster generation advancements for synthetic groundnuts, (c) integration with MABC/MAS/pyramiding for faster development of molecular breeding products and (d) fast-forwarding genomic selection breeding through rapid generation advancement. In summary, the speed breeding has great potential in speeding up the process of genetic population development, accelerated domestication, trait mapping, MAS/MABC and genomic selection breeding in groundnut.

## Modernization of trait discovery and deployment pipeline by adopting new technological interventions

The revolution in genomics and information technologies over the past decade has facilitated development and deployment of different genomics and digital tools in the entire agricultural value chain including crop improvement for achieving faster genetic gains in farmers field (Xu et al. 2017). Multiple research groups located in India, China, USA and Japan have already deployed advanced sequencing technologies for developing reference genomes, sequencing-based trait mapping and successfully developing molecular breeding lines leading to varietal release. There are several such technologies which have great potential in modernizing the crop improvement and agricultural practices, viz*.* (a) sequence-based trait mapping and breeding, (b) low-cost genotyping assays, (c) QC panel, (d) seed chipping and early generation section, (e) speed breeding, (f) genome editing, (g) genomic selection, (h) digitalization, (i) artificial intelligence, (j) faster variety replacement and (k) operational efficiency including mechanization.

The low-cost sequencing technologies and software to analyze even the most complex genomes have brought several orphan crops to the same level playing field as the major crops like rice, wheat and maize. The days are not so far away when every single germplasm line available in the earth will be sequenced, and all the genomic information will be publicly available. In addition to sequencing technologies, the scientific community has witnessed an evolution in availability of different genotyping platforms over the last three decades (Rasheed et al. 2017). Currently, the scientific community is looking for four types of genotyping assays, i.e., (a) high-density genotyping assays with > 20 K SNPs for use in trait mapping; (2) mid-density genotyping assay with 2-5 K SNPs for use in genomic selection and performing background selection; (3) low-density genotyping assay with 100–200 SNPs to be used as quality control (QC) panel; and (4) 10-SNP (associated with traits) panels for performing early generation in for marker-assisted selection (Varshney et al. 2019). In groundnut, high-density genotyping assay with 58 K SNPs has been developed, validated and deployed for different genetic and breeding applications (Pandey et al. 2017a; Clevenger et al. 2017). Also, a 10-SNP panel containing associated SNPs for high oleic acid and foliar disease resistance has been developed and so far > 55,000 groundnut breeding lines have been genotyped under the high-throughput genotyping project (HTPG). Now, the efforts should be made for developing more such 10-SNP panels for other traits and also the mid- and low-density SNP panels for GS and QC applications.

There are several new groundnut varieties which are still struggling to find their place in farmers field as several farmers still grow > 10 years old varieties. In order to achieve higher genetic gains, faster varietal replacement is essential so that the benefits of improved varieties can be harnessed by farmers, consumers and industry (Xu et al. 2017; Varshney et al. 2018). In this context, the rapid cycling (rapid generation advancement) can play important role in faster development of new and improved varieties; and the quality seeds of improved varieties should reach faster to farmers. In summary, the pipeline from laboratory to farmers field should be free from all delays, process and hassles and marker panels can play a critical role in bringing precision and accuracy not only in selection of breeding lines with desirable traits, but also can provide support to entire value chain including adoption tracking and intellectual property rights issues. Furthermore, digitalization should be made for entire operations in entire value chain such as right from selection of parents in breeding, hybridization and selection, evaluation and trials, varietal release, seed production, availability of quality seeds to farmers and cultivation, trading, processing and market of groundnut produce (Varshney et al. 2018). The modernization of entire process, operations and data recording and accessibility will ensure availability of required information and better coordination among different stakeholders on groundnut crop.

## Market and consumer trends

The recent trend in health and wellness is driving innovation in product portfolios being offered by many food manufacturing companies. Groundnuts and nuts in general are becoming increasingly key ingredients and components in nutritional product offering in the market today. These enhancements are not expected to create negative impacts on the edible attributes and highly desired flavor of groundnuts. The development of the high oleic groundnuts in the past few years has dramatically improved shelf life of products made from groundnuts. The oxidative stability of high oleic groundnuts relative to normal oleic types has been well documented. This has been made possible by the development of molecular markers for the high oleic traits, which has enabled development of high oleic groundnuts across the groundnut producing countries. For example, high oleic groundnuts are grown since long in Argentina, Brazil, Australia and USA, while China and India have just started. Most of the groundnuts produced in Asian and African countries go into oil production, compared to Argentina, Brazil and USA, and therefore, the oil content can be enhanced further through genetic selection and breeding.

Groundnuts are one of the highest and cheapest sources of plant-based protein along with an excellent source of folates, niacin and minerals such as magnesium, calcium, copper, antioxidants (including resveratrol). It is also worth mentioning here that groundnuts contain all the essential amino acids necessary for normal body growth and metabolism (Settaluri et al. 2012). Groundnuts are also known to contain reasonable amounts of phenols and polyphenols such as procyanadins. Groundnuts are and could even play a more vital role as a source for introduction of water and fat soluble vitamins into the diets of consumers. They are a major and efficient source of thiamin, niacin, riboflavin and the fat soluble vitamin E (Settaluri et al. 2012). This presents a great opportunity for groundnut to play a major role in nutrition as vital plant protein source. Of course, there is a consumer concern for allergenicity issues in many food products, but a good labeling guideline and use of groundnuts with low allergen content could help manage some of the issues. In this regard, recent studies reported ELISA-based protocol to quantify allergens from groundnut seeds (Pandey et al. 2019b) which was then successfully used for identification of groundnut lines with low allergen content for Ara h1, Ara h2, Ara h3, Ara h6 and Ara h8 (Pandey et al. 2019c). There is substantial variability of these components, thus indicating that the compositions of these beneficial compounds could be enhanced through genetic breeding approaches.

It is important to mention that groundnuts have been playing a major role in the formulation of ready-to-eat therapeutic products. Therefore, groundnut with all the above-mentioned nutritional richness has great potential in becoming the major source of low-cost plant-based protein and other micronutrients in developing countries in Asia and Africa. In coming years, the nutrition-dense delicious groundnut products with desirable flavor compounds are very much on cards which will meet the market and consumer preference.

## Challenges and opportunities

The current groundnut cultivated area and productivity are being greatly challenged by the prevailing biotic/abiotic stresses, inefficient crop management and seed system which has been now further worsened due to fluctuations in weather events such as high-temperature and unpredictable rainfall (time and volume). The diversified use of groundnut for food purpose has increased significantly in recent years, and many more are in pipeline. Each of these specialized groundnut-based products require special feature which needs to be incorporated in newly developed improved varieties, thereby adding further workload to the existing groundnut breeding programs. Therefore, improving the genetics of seeds with fast pace will remain the main challenge before groundnut research community in addition to improving the crop management practices and effective seed system.

This makes it essential to invest our research effort and resources in developing genetic breeding tools and encouraging technology (such as speed breeding, genomic selection and gene editing) that can help in faster development of improved varieties. It is pleasing to note that groundnut has now achieved optimum genomic resources which provides great opportunities together with diverse genetic resources for harnessing the beneficial and favorable alleles in the modern cultivars that can sustain production under climate change scenarios and preference of consumers and traders. These resources provide several opportunities and need strategic research investments for (a) sequencing the entire groundnut germplasm; (b) developing mid-density and low-density marker assays for GS and QC; (c) developing more diagnostic markers for important traits based on fine mapping, gene discovery and function identity; (d) seed-chipping-based high-throughput genotyping in place of leaf-punching method; (e) developing phenotyping protocols for complex traits such as drought and heat tolerance; (f) diversifying the primary gene pool by bringing useful alleles; (g) faster varietal replacement; and (h) digitalization and modernization of entire value chain of groundnut. The groundnut research community so far had remarkable achievements unmatched with any other crop community, and this efficient legacy needs to be continued through collaborations and networking so that the benefits of research can reach the end users and other relevant stakeholders.

## Summary

Groundnut crop has multi-dimensional applications for global population, society and environment. It is important to mention that groundnut being one of the most nutritious crops provides great support to meet daily nutritional requirement as well as fighting the old problem of malnutrition due to affordibility and easy access to low-earning consumers. In the era of global market and trade, only those crop commodities will survive which will be beneficial for farmers and meet the preferences of industry and consumers. The groundnut crop has evolved over the centuries and today has become an essential crop commodity in the food basket for the global population. The groundnut research community not only developed optimum genomic resources (such as genomes) but also deployed the genome information for developing molecular breeding products. The advanced technologies and tools need to be incorporated in all the operations in agriculture for further enhancing the production under changing climate conditions and day-by-day limiting natural resources.
